# Diabetes Mellitus as a Risk Factor for Spontaneous Preterm Birth in Women with a Short Cervix after Ultrasound-Indicated Cerclage

**DOI:** 10.3390/jcm13133727

**Published:** 2024-06-26

**Authors:** Kyong-No Lee, Youngmi Kim, Yeo Kyeong Bae, Jisong Hwang, Yejin Seo, Keun-Young Lee, Jae Jun Lee, Ga-Hyun Son

**Affiliations:** 1 Department of Obstetrics and Gynecology, Chungnam National University Hospital, Daejeon 35015, Republic of Korea; kyongnolee@cnuh.co.kr (K.-N.L.); byk006@cnuh.co.kr (Y.K.B.); 2 Institute of New Frontier Research Team, College of Medicine, Hallym University, Chuncheon 24252, Republic of Korea; kym8389@daum.net; 3 Division of Maternal-Fetal Medicine, Department of Obstetrics and Gynecology, Hallym University College of Medicine, Kangnam Sacred Heart Hospital, Seoul 07441, Republic of Korea; gs3354@hallym.or.kr (J.H.); sp3358@hallym.or.kr (Y.S.); mfmlee@hallym.ac.kr (K.-Y.L.); 4 Departments of Anesthesiology and Pain Medicine, Hallym University College of Medicine, Chuncheon 24252, Republic of Korea

**Keywords:** cervical insufficiency, obesity, spontaneous preterm birth, ultrasound-indicated cerclage

## Abstract

**Background:** Preterm birth (PTB) is a significant challenge in contemporary obstetrics, affecting over one in ten infants worldwide and accounting for 75% of perinatal mortality. Short cervical length during mid-trimester is well known to be associated with an increased risk of spontaneous preterm birth (sPTB). Ultrasound-indicated cerclage (UIC) is recommended to prevent sPTB in women with a short cervix at mid-trimester and a history of sPTB. **Objectives:** This retrospective observational study aimed to examine the impact of diabetes and obesity on the occurrence of sPTB in women who underwent UIC due to mid-trimester cervical shortening. **Methods/Results:** The analysis revealed that cervical length at the time of operation, preoperative erythrocyte sedimentation rate levels, and diabetes were independent risk factors for sPTB. Additionally, the presence of diabetes, particularly when combined with obesity, significantly elevated the risk of sPTB. Women with pregestational diabetes or those requiring insulin treatment had a higher propensity for preterm delivery compared to those with gestational diabetes managed through diet control alone. **Conclusions:** These findings emphasize the importance of considering maternal metabolic factors, such as diabetes and obesity, in women with a short cervix when planning for UIC and highlight the crucial role of optimizing maternal glucose control and weight management in reducing the risk of sPTB.

## 1. Introduction

Preterm birth (PTB), defined as delivery before 37 weeks of gestation, is a significant challenge in contemporary obstetrics that affects over one in ten infants worldwide, accounting for 75% of perinatal mortality. Neonates born preterm face increased risks of long-term morbidity, including neurodevelopmental impairments, as well as respiratory and gastrointestinal complications [1]. Women at an elevated risk for PTB have been identified through maternal clinical risk factors or obstetric history; however, the effectiveness of this method in predicting PTB remains limited. A pivotal predictor of spontaneous preterm birth (sPTB) is short cervical length, as determined by mid-trimester transvaginal ultrasonography [2]. Research indicates a correlation between cervical length and the risk of sPTB: approximately 0.4% at 6.0 cm, 15.2% at 2.5 cm, 22.8% at 1.5 cm, and 57% at 0.5 cm. This risk increases the early detection of a short cervix in the context of preterm birth prevention [3]. To mitigate sPTB in women with mid-trimester cervical shortening, a variety of strategies have been employed. These include progesterone administration, cerclage, cervical pessary, and lifestyle modifications such as quitting of smoking and nutritional supplements.

Specifically, in singleton pregnancies, cervical cerclage for women with a history of sPTB and a cervical length less than 2.5 cm before 24 weeks has been shown to decrease perinatal morbidity and mortality [4,5]. As a result, ultrasound-indicated cerclage (UIC) is now recommended for these patients [6,7]. However, in cases of singleton pregnancies with no prior sPTB history and a mid-trimester cervical length below 2.5 cm, UIC has not been proven to significantly prevent preterm delivery or improve neonatal outcomes [8]. Nonetheless, recent research indicates that for women with a very short cervix (less than 1.0 cm) who have no sPTB history, UIC can extend the duration of pregnancy and reduce the risk of earlier delivery post-diagnosis, compared to those not receiving UIC [9]. Moreover, occurrences of late preterm birth (34–37 weeks) are less frequent among patients who undergo UIC, suggesting its consideration for patients with an extremely short cervix and no prior sPTB history [10]. Despite the inherent high risks, some successfully carry pregnancies to term after cerclage operation, while others may experience preterm or periviable deliveries. Predictive factors for successful outcomes in women undergoing UIC are still unclear and likely involve multiple factors [11,12].

In recent years, the increasing prevalence of diabetes during pregnancy and obesity, often co-existing with diabetes, has garnered significant attention due to their potential impact on pregnancy outcomes, including the risk of sPTB. Although it is known that hypertensive complications, abnormal fetal growth, and increased urinary tract infections, which are more commonly associated with diabetes, lead to an increase in medically indicated PTB [13,14,15], Köck et al. reported that diabetes affects the length of gestation and increases the incidence of sPTB as well [16]. Moreover, the risk of sPTB increases with higher levels of pregnancy glycemia [17,18,19]. While some studies have identified pre-pregnancy obesity as a risk factor for sPTB, others have reported null or even inverse associations [20,21]. This variability in the correlation between obesity and the likelihood of sPTB may be attributed to differences in race or ethnicity. However, when obesity and diabetes coexist, most studies consistently reported that the incidence of both medically indicated preterm births and sPTB increases significantly more than when either factor is present alone [22]. However, to date, there have been few reports on how maternal diabetes or obesity affect the occurrence of sPTB in women with short cervix at mid-trimester, a high-risk group of sPTB. Therefore, this study aimed to examine the impact of diabetes and obesity on the occurrence of sPTB in patients who underwent UIC due to mid-trimester cervical shortening.

## 2. Materials and Methods

### 2.1. Study Design and Population

**Figure 1:**
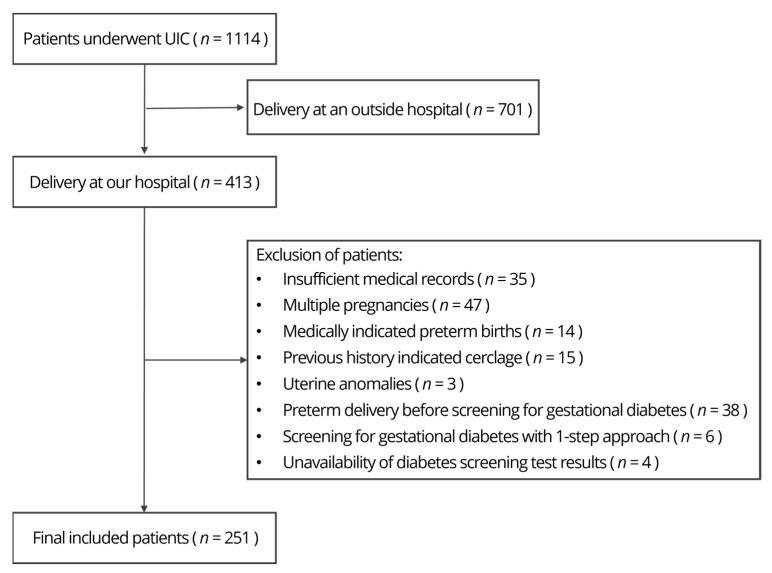
Flowchart of the study.

This retrospective cohort study was conducted by searching a comprehensive database of women admitted to our institution. The study was approved by the Institutional Review Board Ethics Committee of the Hallym University Kangnam Sacred Heart Hospital (IRB protocol code 2022-09-019). Patients who underwent UIC between 15 and 26 weeks of gestation and delivered were recruited at the Hallym University Kangnam Sacred Heart Hospital from January 2014 to September 2023. Figure 1 shows the enrollment flowchart. The inclusion criteria were as follows: (1) UIC performed due to a cervical length of <2.5 cm, (2) singleton pregnancy, and (3) delivery at our institution. Exclusion criteria included multiple pregnancies, fetuses with major anomalies, uterine anomalies, ruptured membranes, vaginal bleeding, regular uterine contractions, history of cervical conization or loop electrosurgical excision procedures, prophylactic cerclage, or medically indicated PTB, preterm delivery before screening for gestational diabetes, unavailability of diabetes screening test results, and the one-step approach using a 2-h, 75 g oral glucose tolerance test for the diagnosis of GDM.

Before performing UIC, cervical length was evaluated using transvaginal ultrasonography. The probe was placed at the anterior fornix of the vagina with an empty bladder to avoid any pressure that could distort the cervix. Cervical measurement is obtained by placing calipers at the external and the internal os, recording the shortest distance observed during dynamic cervical changes. Additionally, the extent of cervical funneling was documented. Before the UIC, maternal blood tests included analyses of serum white blood cell (WBC) count, erythrocyte sedimentation rate (ESR), and C-reactive protein (CRP) levels, with a CRP level above 5.0 mg/L indicating a high reading.

Data on patients, including demographic and obstetric history, was sourced from electronic medical records. Measurements of maternal height and weight were routinely taken at outpatient visits and during hospital admissions for procedures or delivery. Pre-pregnancy weight was noted from patient self-reports. The BMI was calculated by a person’s weight in kilograms divided by the square of height in meters. According to the criteria for diagnosing obesity in adults in South Korea, the BMI categorization was as follows: a BMI < 18.5 kg/m^2^ was classified as underweight, 18.5–24.9 kg/m^2^ as normal weight, 25.0–29.9 kg/m^2^ as class I obesity, ≥30 kg/m^2^ as class II obesity [23]. Pregestational diabetes is defined as diabetes that existed in a woman before she became pregnant. GDM was diagnosed using a two-step method. First, a 1-h, 50 g glucose challenge test was used as a screening test (threshold plasma glucose 140 mg/dL). If the screening test was positive, it was followed by a diagnostic 3-h oral glucose tolerance test. The diagnosis was confirmed when any two or more values in the 100 g, 3-h test met or exceeded the Carpenter and Coustan criteria [24].

### 2.2. Procedure

UIC was performed in patients with a prior history of PTB presenting with a cervical length of less than 2.5 cm between 15 and 26 weeks of gestation. Furthermore, UIC was performed on patients exhibiting a cervical length of less than 1.0 cm, independent of their previous sPTB history. For patients without a history of sPTB and who had a cervical length ranging from 1.0 cm to 2.5 cm during mid-trimester sonography, vaginal progesterone was prescribed. While using vaginal progesterone and conducting follow-up observations, if cervical shortening worsened without clinical symptoms such as uterine contractions or vaginal bleeding, we proceeded with UIC. All cerclage procedures used the McDonald Technique, employing a single 5 mm Mersilene tape suture in a purse-string fashion. A Uniconcave balloon device was used during cerclage procedures in patients exhibiting a ripe and short cervix to prevent iatrogenic rupture of fetal membranes [25]. Tocolytics were administered postoperatively if uterine contractions were noted on the tocogram after the cerclage operation. Cephalosporin antibiotics were given prophylactically prior to UIC and extended for three days after the surgery. Typically, patients were discharged on the third day post-cerclage, unless they exhibited additional symptoms. Follow-up appointments were scheduled two weeks after post-discharge at the outpatient clinic, with routine follow-ups planned if no cervical shortening was observed. Vaginal progesterone (200 mg) was administered post-operation until 36 weeks of gestation. Repeat cerclage was performed in cases of membrane prolapsed beyond the external os before 26 weeks of gestation post-cerclage without clinical symptoms such as preterm labor, vaginal bleeding, or ruptured membranes.

### 2.3. Statistical Analysis

Data analysis was performed using SPSS software (version 27.0; IBM Corp., Armonk, NY, USA). Univariate analysis utilized a range of statistical tests deemed suitable, including Student’s *t*-test, Mann–Whitney U test, Kruskal–Wallis test, chi-square test with post hoc tests (custom tables), and Fisher’s exact test, all of which were conducted with two tails. Variables that demonstrated significant links with sPTB during univariate analysis were then analyzed using multivariate logistic regression to assess their independent relationships with sPTB, controlling for additional variables. Statistical significance was set at *p* < 0.05.

## 3. Results

In this study, 251 patients were included. The demographic and clinical characteristics of the participants are shown in Table 1. The mean gestational age at the time of cerclage operation was 22.5 ± 2.9 weeks, with an average cervical length of 14.9 ± 6.0 mm. The mean gestational age at delivery was recorded as 35.2 ± 4.3 weeks, and 126 (50.2%) women underwent preterm delivery. Out of a total of 251 patients, 64 (25.5%) were obese, 23 (9.1%) had class II obesity, and 37 were diagnosed with GDM or pregestational diabetes.

A comparative analysis of the preterm delivery group and full-term delivery group according to gestational age at delivery is presented in Table 2. There was no significant difference between the two groups in terms of maternal age or obstetric history. However, a significant difference was observed in pre-pregnancy BMI, as well as in the frequency of obesity and diabetes. Additionally, in the preterm delivery group, the cervical length at the time of the cerclage operation was significantly shorter, and the levels of inflammatory markers, such as ESR and WBC count, were higher.

These confounding variables were selected according to univariate analysis as risk factors for preterm delivery after cerclage operation. We applied a multivariable logistic regression analysis with confounding variables, including maternal age, diabetes, cervical length at cerclage operation, and preoperative ESR. In this model, preoperative cervical length, preoperative ESR level, and diabetes were independently associated with an increased risk of sPTB after UIC (Table 3). Notably, diabetes was a significant risk factor for sPTB (OR 2.41, 95% confidence interval, 1.07–5.43, *p* = 0.008).

In a comparative analysis between the diabetes and non-diabetes groups, there were no differences in obstetric history and gestational weeks at cerclage operation. However, the diabetes group exhibited a greater prevalence of obesity, and levels of ESR, CRP, and WBC were higher in the diabetes group. Moreover, while preoperative cervical length did not differ between the two groups, the frequency of a presence of cervical funneling was higher in the diabetes group, and repeat cerclage was more common in this group. When analyzing pregnancy outcomes, the diabetes group was associated with earlier gestational weeks of delivery and a significantly higher incidence of sPTB compared with the normal group (Table 4). In the diabetes group, sPTB occurred in 26 out of 37 cases (70.3%), whereas in the non-diabetes group, it occurred in 100 out of 214 cases (46.7%), moreover, this trend intensified with earlier gestational weeks of preterm delivery. For extremely preterm birth (less than 28 weeks), the diabetes group had a rate of 9 out of 37 cases (24.3%), while in the normal group, only 10 out of 214 cases (4.7%) experienced extremely preterm birth (*p* < 0.001).

Next, an analysis was conducted to examine which factors in the diabetes group are associated with sPTB. The results showed that among the 37 women with diabetes, 26 experienced preterm delivery. Within the diabetes group, a shorter cervical length at the time of cerclage operation or higher levels of inflammatory markers such as ESR and CRP were associated with sPTB. Additionally, although not statistically significant, there was an association between sPTB and Class II obesity, where 11 out of 12 severely obese diabetes women experienced sPTB. Furthermore, while HbA1c levels did not differ between the preterm delivery group and the full-term delivery group, there was a noticeable trend towards a higher risk of sPTB in the pregestational diabetes group compared to those with GDM (10/11 (90.9%) vs. 16/26 (61.5%), *p* = 0.119) (Supplementary Table S1). This trend was particularly evident when the severity of diabetes required insulin use as opposed to management through diet control alone (Table 5).

## 4. Discussion

The results of this study indicate that cervical length at the time of operation, preoperative ESR levels, and diabetes were independently associated with an increased risk of sPTB following UIC. The diabetes group exhibited a higher prevalence of obesity and elevated levels of ESR, CRP, and WBC compared to the non-diabetes group. Additionally, the frequency of cervical funneling was higher in the diabetes group, and repeat cerclage was more common. Moreover, in the diabetes group, gestational age at delivery was earlier, and sPTB occurred more frequently compared to the non-diabetes group. The disparity in preterm birth frequency between the diabetes and non-diabetes groups was more pronounced with earlier gestational weeks. Furthermore, within the diabetes group, women with Class II obesity had a stronger association with preterm delivery. Notably, those with pregestational diabetes or requiring insulin use were more likely to experience preterm delivery compared to those with GDM or those managed through diet control.

Several studies have been reported so far analyzing the risk factors for preterm delivery after cerclage operation. These previous studies have reported that factors such as nulliparity, earlier gestational age at the time of the cerclage procedure, a history of sPTB, advanced cervical dilation, prolapse of the fetal membranes into the vagina, and elevated CRP levels are associated with the success of emergency cerclage [26,27,28,29]. However, research analyzing the risk of sPTB following UIC or physical examination-indicated cerclage has primarily focused on factors such as cervical status or gestational age at the time of cerclage placement, and markers indicative of intra-uterine inflammation or infection in maternal blood or amniotic fluid. Recently, there has been a notable increase in the prevalence of obesity and diabetes complicating pregnancy, leading to an increase in research interest in their potential influence on PTB. Maternal factors linked with diabetes are becoming more widespread, largely attributed to the escalating rates of overweight and obesity among women, both recognized risk factors for the onset of type 2 diabetes and GDM. Additionally, there’s a trend towards older maternal age at the time of first pregnancy, further contributing to this landscape [30]. Nevertheless, the precise connection between pre-pregnancy obesity, diabetes, and the risk of sPTB continues to be a subject of debate and contention [16,21,31]. Lee et al. reported that, compared to the normoglycemic group, the GDM group exhibited significantly higher rates of PTB [32]. Another study also demonstrated that both pregestational diabetes and GDM are associated with a higher incidence of PTB [33]. However, recent systematic reviews have reported that, compared to normal-weight women, women with obesity are not at an increased risk of PTB [34]. Conversely, recent systematic reviews suggest that women with a BMI of 35 kg/m^2^ or higher are at greater risk of experiencing very preterm birth (before 32 weeks) and moderate preterm birth (between 32 and 36 weeks) [31,35]. Further, several studies have underscored an elevated risk of extremely preterm birth (before 28 weeks) in women with a BMI of 30 kg/m^2^ or higher [35,36,37,38]. Shaw et al., in a detailed population-based study, found an increased risk of preterm birth before 27 weeks among mothers with severe obesity, noting that this risk varies by ethnicity and parity [39]. While research results on the risk of sPTB in mothers with obesity are inconsistent, but most studies have consistently demonstrated that the combination of obesity and diabetes is associated with a higher risk of preterm delivery compared to each factor alone [16,33,34].

Insulin resistance, hyperglycemia, adipose tissue dysfunction, oxidative stress and other factors accompanying diabetes are known contributors to chronic inflammation, which can alter the normal inflammatory processes necessary for cervical ripening [40,41]. The altered cytokine environment in diabetic pregnancies may affect cervical remodeling processes like softening, shortening, and dilation [42,43]. Our study findings reveal elevated levels of nonspecific chronic inflammation markers, such as ESR, in women with diabetes. Although cervical length didn’t differ between diabetes and non-diabetes groups in our study, the increased frequency of cervical funneling and repeat cerclage in the diabetes group suggests a potential influence of diabetes on the severity of cervical insufficiency, thereby elevating the risk of preterm delivery. We observed a significant correlation between ESR and BMI (*p* < 0.001), and ESR was particularly elevated in the obese and Class II obesity groups. Additionally, individuals with diabetes had notably higher ESR levels than those without diabetes. Moreover, pregestational diabetes and insulin use further elevated ESR levels within the diabetes group (*p* = 0.028, *p* = 0.026, respectively). Hence, our study implies that while ESR may be a nonspecific marker in patients with a short cervix post-cerclage operation, it deserves more attention as a marker associated with preterm birth occurrence in women with a short cervix. Moreover, inadequate glycemic control in diabetic pregnancies can lead to the formation of advanced glycation end products, which are reported to interfere with the normal cross-linking of collagen fibers [44,45,46]. This interference can compromise cervical tissue integrity, potentially leading to premature cervical ripening. Our results demonstrating the increased risk of PTB in the diabetes group, and the exacerbation of this trend with the severity of diabetes, further support these findings. A recent large cohort study revealed that maternal obesity, especially when combined with diabetes, significantly increased the likelihood of PTB. Insulin-treated diabetes, in particular, posed the highest risks, notably associated with an elevated likelihood of very and extremely preterm births [18]. This finding aligns with our results.

This study had several limitations. First, because most participants were of Korean ethnicity, we adopted a BMI of 25 kg/m^2^ as the cut-off for distinguishing between obese and non-obese patients. Therefore, our findings should be interpreted with caution when considering Western populations, where a BMI of 30 kg/m^2^ or higher is classified as obese. Second, our analysis was limited to patients who delivered at our institution, enabling clear verification of birth records. This approach may yield different outcomes from studies that include births in community hospitals or other settings. Third, our institution houses a level III neonatal intensive care unit, attracting expectant women with symptoms of PTB or at higher risk of sPTB, which increases the probability of them giving birth at our facility. On the other hand, patients showing no PTB symptoms post-UIC often opt to deliver at local community hospitals, and thus were not included in our study, potentially impacting our findings. This could explain the higher incidence of PTB seen in our research compared to others, suggesting possible selection bias. Fourth, the diabetes group was relatively small, and due to the retrospective study design, detailed information on maternal serum markers, such as insulin resistance, diabetes control, and markers related to obesity, was limited. Moreover, another limitation of this study is the grouping of GDM and pregestational diabetes into the same category. Although there is a continuum of insulin resistance in both conditions, they are distinct in terms of pathophysiology, clinical management, and perinatal complications. However, the insufficient sample sizes of each group in our study necessitated their combination for analysis to examine the overall impact of diabetes on the occurrence of preterm birth. Future studies with larger sample sizes are needed to separately analyze these two groups and their associations with other clinical factors.

Our study is significant in the context of examining diabetes as a risk factor for sPTB in women with a short cervix who have undergone UIC. Although there have been numerous discussions about the correlation between GDM, obesity, and sPTB, our research is notable for focusing specifically on the outcomes of diabetes and obesity in a cohort that received UIC. This targeted analysis provides valuable insights into the impact of these factors within this specific population.

## 5. Conclusions

This study demonstrated that cervical length at the time of operation, ESR levels, and diabetes were independent risk factors for sPTB following UIC in women with short cervical lengths in the mid-trimester. Moreover, diabetes, particularly when combined with obesity, significantly elevated the risk of sPTB. Notably, women with pregestational diabetes or those requiring insulin treatment exhibited a higher propensity for preterm delivery compared to those with GDM managed through diet control alone. These findings underscore the importance of considering maternal metabolic factors such as diabetes and obesity in women with short cervixes when considering UIC. It is crucial to recognize that optimizing maternal glucose control and weight management can significantly affect the risk of sPTB.

## Figures and Tables

**Table 1:** Demographic and clinical characteristics of the study population.

Characteristic	Total Patients (*n* = 251)
Age (years)	34.0 ± 4.2
Primipara	133 (53.0)
Race/Ethnicity	
Korean	234 (93.2)
Chinese	11 (4.4)
Southeast Asian	4 (1.6)
Caucasian	2 (0.8)
History of full-term birth	88 (35.1)
History of preterm birth	36 (14.3)
Height (cm)	161.1 ± 5.5
Pre-pregnancy body weight (kg)	59.4 ± 13.1
Pre-pregnancy BMI (kg/m^2^)	22.9 ± 4.6
Obesity (BMI ≥ 25 kg/m^2^)	64 (25.5)
Class II Obesity (BMI ≥ 30 kg/m^2^)	23 (9.1)
Pregestational or Gestational diabetes	37 (14.7)
Gestational diabetes	26 (10.4)
Hypertensive disease	7 (2.7)
Gestational week at operation (weeks)	22.5 ± 2.9
Preoperative cervical length (mm)	14.9 ± 6.0
Preoperative cervical length < 10 mm	61 (24.3)
Presence of cervical funnel	175 (69.7)
Inflammatory serum marker results	
ESR (mm/h)	35.4 ± 17.6
CRP (mg/L)	5.9 ± 11.0
WBC count (/μL)	9632.1 ± 2342.1
Neurtrophil (%)	76.4 ± 5.5
Repeat cerclage	14 (5.5)
Gestational age at delivery (weeks)	35.2 ± 4.3
sPTB before 37 weeks	126 (50.2)
sPTB before 34 weeks	72 (28.7)
sPTB before 28 weeks	19 (7.6)

Values are expressed as mean ± SD or *n* (%). BMI, body mass index; ESR, erythrocyte sedimentation rate; CRP, C-reactive protein; WBC, white blood cell; sPTB, spontaneous preterm birth.

**Table 2:** Comparison of clinical characteristics between the preterm delivery group and full-term delivery group.

Characteristic	Preterm Group (*n* = 126)	Full-Term Group (*n* = 125)	*p* Value
Age (years)	33.7 ± 4.2	34.3 ± 4.1	0.253
History of full-term birth	39 (31.0)	49 (39.7)	0.171
History of preterm birth	18 (14.3)	18 (14.4)	0.979
Height (cm)	160.7 ± 5.8	161.4 ± 5.3	0.280
Pre-pregnancy body weight (kg)	61.2 ± 15.4	57.5 ± 9.9	0.022
Pre-pregnancy BMI (kg/m^2^)	23.6 ± 5.1	22.1 ± 3.7	0.007
Obesity (BMI ≥ 25 kg/m^2^)	40 (31.7)	24 (19.2)	0.023
Class II Obesity (BMI ≥ 30 kg/m^2^)	17 (13.5)	6 (4.8)	0.017
Pregestational or Gestational diabetes	26 (20.6)	11 (8.8)	0.008
50 g GCT test high	50 (39.7)	36 (28.8)	0.069
Gestational week at operation (weeks)	22.5 ± 2.8	22.5 ± 2.9	0.996
Preoperative cervical length (mm)	13.7 ± 6.0	16.2 ± 5.5	0.001
Preoperative cervical length < 10 mm	42 (33.3)	19 (15.2)	0.001
Presence of cervical funnel	93 (73.8)	82 (65.6)	0.157
Inflammatory serum marker			
ESR (mm/h)	38.9 ± 18.9	31.6 ± 15.0	0.001
CRP (mg/L)	7.1 ± 9.0	4.6 ± 12.6	0.079
WBC count (/μL)	9919.0 ± 2616.7	9335.8 ± 1955.0	0.047
Neurtrophil (%)	76.8 ± 5.7	76.0 ± 5.2	0.252
Repeat cerclage	10 (7.9)	4 (3.2)	0.167
Gestational age at delivery (weeks)	32.0 ± 3.8	38.4 ± 1.0	<0.001

Values are expressed as mean ± SD or *n* (%). BMI, body mass index; ESR, erythrocyte sedimentation rate; CRP, C-reactive protein; WBC, white blood cell.

**Table 3:** Result of the multivariate logistic regression analysis.

Clinical Characteristics	Multivariate Analysis		
	Odds Ratio	95% Confidence Interval	*p* Value
Maternal age (years)	0.96	0.90–1.02	0.163
Diabetes mellitus	2.41	1.07–5.43	0.008
Preoperative cervical length (mm)	0.92	0.88–0.97	0.001
Preoperative ESR (mm/h)	1.03	1.01–1.04	0.002

ESR, erythrocyte sedimentation rate.

**Table 4:** Comparison of patients between the diabetes group and non-diabetes group.

Characteristic	Diabetes (*n* = 37)	Non-Diabetes (*n* = 214)	*p* Value
Age (years)	35.4 ± 3.9	33.8 ± 4.1	0.037
History of full-term birth	10 (27.0)	78 (36.4)	0.267
History of preterm birth	7 (18.9)	29 (13.6)	0.390
Height (cm)	161.7 ± 5.8	160.9 ± 5.5	0.438
Pre-pregnancy body weight (kg)	72.7 ± 17.6	57.1 ± 10.6	<0.001
Pre-pregnancy BMI (kg/m^2^)	27.6 ± 5.7	22.0 ± 3.7	<0.001
Obesity (BMI ≥ 25 kg/m^2^)	27 (73.0)	37 (17.3)	<0.001
Class II Obesity (BMI ≥ 30 kg/m^2^)	12 (32.4)	11 (5.1)	<0.001
Gestational week at operation (weeks)	22.1 ± 3.3	22.6 ± 2.8	0.362
Preoperative cervical length (mm)	14.9 ± 5.7	14.9 ± 5.9	0.975
Preoperative cervical length < 10 mm	8 (21.6)	53 (24.8)	0.836
Presence of cervical funnel	31 (83.8)	144 (67.3)	0.044
Inflammatory serum marker results			
ESR (mm/h)	44.2 ± 23.5	33.7 ± 15.7	0.012
CRP (mg/L)	13.4 ± 22.0	4.5 ± 6.7	0.020
WBC count (/μL)	10,514.6 ± 2091.9	9475.4 ± 2333.2	0.012
Neurtrophil (%)	76.3 ± 5.8	76.4 ± 5.4	0.915
Repeat cerclage	5 (13.5)	9 (4.2)	0.039
Gestational age at delivery (weeks)	32.6 ± 5.7	35.7 ± 3.8	0.003
sPTB before 37 weeks	26 (70.3)	100 (46.7)	0.008
sPTB before 34 weeks	18 (48.6)	54 (25.2)	0.004
sPTB before 28 weeks	9 (24.3)	10 (4.7)	<0.001

Values are expressed as mean ± SD or *n* (%). BMI, body mass index; ESR, erythrocyte sedimentation rate; CRP, C-reactive protein; WBC, white blood cell; sPTB, spontaneous preterm birth.

**Table 5:** Comparison of clinical characteristics between the preterm delivery group and full-term delivery group in women with diabetes mellitus.

Characteristic	Preterm Group (*n* = 26)	Full-Term Group (*n* = 11)	*p* Value
Age (years)	35 (31–38)	35 (33–40)	0.852
History of full-term birth	5 (19.2)	5 (45.5)	0.125
History of preterm birth	7 (26.9)	0	0.080
Height (cm)	164.0 (158–166.4)	159.7 (157–160)	0.951
Pre-pregnancy body weight (kg)	77 (65.5–86.3)	61 (52–68)	0.229
Pre-pregnancy BMI (kg/m^2^)	28.3 (25.5–33.1)	25.3 (20.8–26.6)	0.114
Obesity (BMI ≥ 25 kg/m^2^)	21 (80.8)	6 (54.5)	0.125
Class II Obesity (BMI ≥ 30 kg/m^2^)	11 (42.3)	1 (9.1)	0.064
Gestational week at operation (weeks)	22.3 (18.9–23.8)	23.2 (20.6–25.2)	0.753
Preoperative cervical length (mm)	13.5 (9.6–19.3)	15.9 (13.0–23.0)	0.020
Preoperative cervical length < 10 mm	7 (26.9)	1 (9.1)	0.391
Presence of cervical funnel	22 (84.6)	9 (81.8)	1.000
Pregestational diabetes	10 (38.5)	1 (9.1)	0.119
Gestational diabetes mellitus	16 (61.5)	10 (90.9)	0.119
HbA1c-NGSP	5.6 (5.2–6.0)	5.1 (5.1–5.9)	0.915
Insulin use	14 (53.8)	2 (18.2)	0.071
Inflammatory serum marker results			
ESR (mm/h)	39.0 (24.5–69.0)	41.0 (20.0–64.0)	0.004
CRP (mg/L)	6.1 (4.0–10.5)	8.8 (4.7–41.8)	0.004
WBC count (/μL)	11,115.0 (8835.0–11,907.5)	9590.0 (9250.0–10,520.0)	0.658
Neurtrophil (%)	77.8 (74.7–80.9)	77.3 (69.9–80.0)	0.411
Repeat cerclage	4 (15.4)	1 (9.1)	0.167
Gestational age at delivery (weeks)	30.9 (27.1–34.4)	38.0 (37.6–39.1)	<0.001

Values are described in median (interquartile range) or *n* (%). BMI, body mass index; ESR, erythrocyte sedimentation rate; CRP, C-reactive protein; WBC, white blood cell; HbA1c-NGSP, Glycated haemoglobin-National Glycohemoglobin Standardization Program.

## Data Availability

The data presented in this study are available on request from the corresponding author. The data are not publicly available due to patients’ privacy.

## Supplementary Materials

The following supporting information can be downloaded at: https://www.mdpi.com/article/10.3390/jcm13133727/s1, Supplementary Table S1: Comparison of clinical characteristics between the gestational diabetes group and Pregestational diabetes group.
