# Supplementary material for: Diabetes Mellitus as a Risk Factor for Spontaneous Preterm Birth in Women with a Short Cervix after Ultrasound-Indicated Cerclage
Source: J Clin Med. 2024 Jun 26;13(13):3727. doi: 10.3390/jcm13133727 (PMC11242270; doi:10.3390/jcm13133727)
Supplement: Supplementary file 1 [file jcm-13-03727-s001.zip › jcm-3050049-supplementary.pdf]

Supplementary Table S1. Comparison of clinical characteristics between the gestational diabetes group and Pregestational diabetes group

Characteristic	Gestational diabetes (n = 26)	Pregestational diabetes (n = 11)	p Value
Age (years)	35 (33.0-39.0)	35 (30.8-37.0)	0.781
History of full-term birth	6 (23.1)	4 (36.4)	0.442
History of preterm birth	4 (15.4)	3 (27.3)	0.403
Pre-pregnancy body weight (kg)	67 (57.3-78.8)	82 (67.8-109.3)	0.023
Pre-pregnancy BMI (kg/m^2)	26.4 (22.0-30.4)	30.6 (27.0-37.4)	0.065
Obesity (BMI ≥ 25 kg/m^2)	18 (69.2)	9 (81.8)	0.688
Class II Obesity (BMI ≥ 30 kg/m^2)	7 (26.9)	5 (45.5)	0.443
Gestational week at operation (weeks)	22.5 (19.7-25.2)	21.4 (17.5-23.9)	0.181
Preoperative cervical length (mm)	13.5 (9.5-21.2)	14.1 (11.8-21.8)	0.883
Preoperative cervical length < 10 mm	7 (26.9)	1 (9.1)	0.391
Presence of cervical funnel	23 (88.5)	8 (72.7)	0.335
HbA1c-NGSP	5.5 (5.1-5.9)	5.7 (5.3-7.2)	0.132
Insulin use	5 (19.2)	11 (100)	<0.001
Inflammatory serum marker results			
ESR (mm/hr)	45.5 (24.8-68.3)	32.5 (24.0-48.8)	0.204
CRP (mg/L)	6.7 (4.6-11.5)	6.7 (4.0-14.0)	0.780
WBC count (/μL)	10425.0 (9285.0-11865.0)	10945.0 (8830.0-11742.5)	0.961
Neurtrophil (%)	77.8 (73.6-80.6)	79.3 (75.5-81.3)	0.366
Repeat cerclage	3 (11.5)	2 (18.2)	0.623
Gestational age at delivery (weeks)	34.8 (27.9-38.0)	33.1 (26.1-35.0)	0.170
sPTB before 37 weeks	16 (61.5)	10 (90.9)	0.119

Values are described in median (interquartile range) or n (%). BMI, body mass index; ESR, erythrocyte sedimentation rate; CRP, C-reactive protein; WBC, white blood cell; sPTB, spontaneous preterm birth; HbA1c-NGSP, Glycated haemoglobin-National Glycohemoglobin Standardization Program.
